# The role of talin2 in breast cancer tumorigenesis and metastasis

**DOI:** 10.18632/oncotarget.22449

**Published:** 2017-11-06

**Authors:** Liqing Li, Xiang Li, Lei Qi, Piotr Rychahou, Naser Jafari, Cai Huang

**Affiliations:** ^1^ Markey Cancer Center, University of Kentucky, Lexington, KY 40506, USA; ^2^ Department of Pharmacology and Nutritional Sciences, University of Kentucky, Lexington, KY 40506, USA

**Keywords:** talin2, cell migration, invasion, tumor growth, metastasis

## Abstract

Recent studies show that talin2 has a higher affinity to β-integrin tails and is indispensable for traction force generation and cell invasion. However, its roles in cell migration, cancer cell metastasis and tumorigenesis remain to be determined. Here, we used MDA-MB-231 human breast cancer cells as a model to define the roles of talin2 in cell migration, invasion, metastasis and tumorigenesis. We show here that talin2 knockdown (KD) inhibited cell migration and focal adhesion dynamics, a key step in cell migration, and that talin2 knockout (KO) inhibited cell invasion and traction force generation, the latter is crucial for cell invasion. Re-expression of talin2^WT^ in talin2-KO cells restored traction force generation and cell invasion, but that of talin2^S339C^, a β-integrin-binding deficient mutant, did not. Moreover, talin2 KO (or KD) suppressed tumorigenesis and metastasis in mouse xenograft models. However, surprisingly, re-expression of talin2^WT^ in talin2-KO cells did not rescue tumorigenesis. Thus, talin2 is required for breast cancer cell migration, invasion, metastasis and tumorigenesis, although exogenous expression of high levels of talin2 could inhibit tumorigenesis.

## INTRODUCTION

Integrin-mediated mechanic signals regulate tumor cell migration, invasion, growth and metastasis [1–6]. Integrins are heterodimers, comprising of α (alpha) and β (beta) subunits [7]. Talin, a large focal adhesion protein, binds to the β subunits, thus activating integrin and regulating a variety of physiological and pathological processes [8–11].

There are two talin genes, *Tln1* and *Tln2*, encoding talin1 and talin2, respectively [12]. Talin1 is essential for cell migration, invasion, tumor growth and metastasis [13–16]. Talin1 regulates focal adhesion (FA) dynamics [13, 17, 18] and invadopodium formation [19], key steps in cell migration and invasion [20–22]. Talin1 also mediates calpain-induced FA disassembly [17, 18]. Along with our collaborators, we have shown that talin1 phosphorylation by Cdk5 regulates FA dynamics, integrin activation, cell migration and invasion [13, 14]. It recruits the moesin-NHE1 complex to modulate pH at invadopodia, consequently governing invadopodium stability and matrix degradation [19]. It is also required for the generation of mechanical force [23], a driver of cell migration and invasion. However, the role of talin2 in these processes are less understood. It has been reported that depletion of talin2 does not influence β1-integrin activation [14], and that talin2-ablated mice are viable [24]. Thus, it was originally thought that talin2 functioned redundantly with talin1.

However, we demonstrated that talin2 had a stronger binding to β integrin tails than talin1 [25, 26]. Talin2 Ser339 is largely responsible for this affinity difference and substitution of Ser339 with Leu disrupted the binding of talin2 to β1-integrin tails. We also demonstrated that talin2 is localized at invadopodia and that a strong talin2-integrins interaction is required for traction force generation and invadopodium formation and cell invasion [25].

In the present study, we used highly aggressive breast cancer cell line MDA-MB-231 cells as a model to investigate the role of talin2 in cell migration, invasion, tumor growth and metastasis. We found that talin2 plays a crucial role in cell migration, invasion, tumorigenesis and metastasis, although exogenous expression of high levels of talin2 could suppress tumorigenesis.

## RESULTS

**Figure 1:**
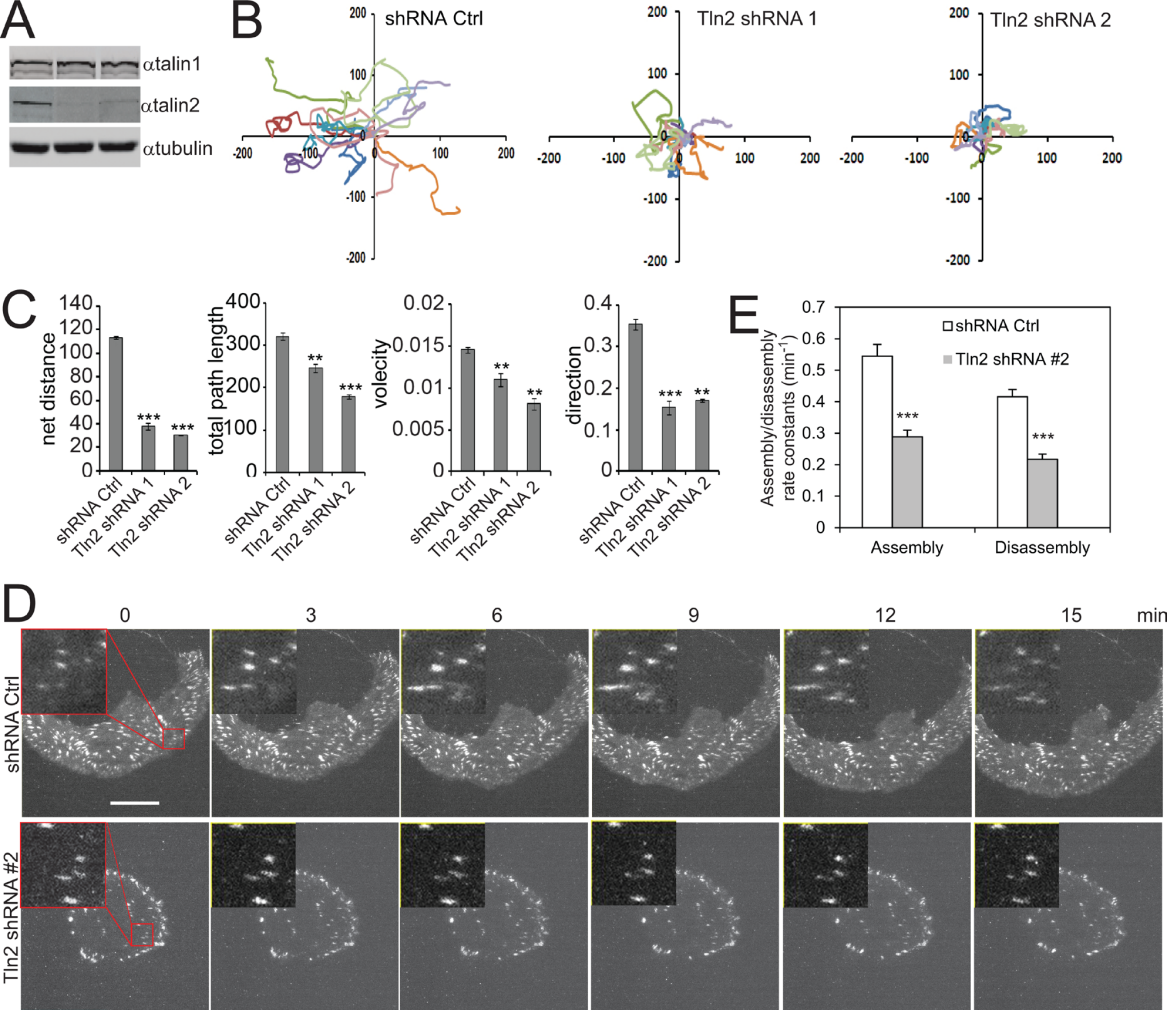
Talin2 is essential for cell migration and FA dynamics in MDA-MB-231 cells (**A**) MDA-MB-231 cells were infected with lentiviruses that express talin2 shRNAs or empty pLKO.1 vector (shRNA control). (**B**) Migration tracks of 10 MDA-MB-231 cells that stably express talin2 shRNAs or empty pLKO.1 vector. (**C**) Statistic results of velocity, total path length, net distance, and directionality of the cells that stably express shRNA control or talin2 shRNA. The data are expressed as mean + S.E.M. of more than 50 cells from three independent experiments. ^*^P < 0.05, ^**^P < 0.01, ^***^P < 0.001 compared to control cells. (**D**) MDA-MB-231 cells that stably express mDsRed-paxillin were infected with lentiviruses that express talin2 shRNA or empty pLKO.1 vector. The cells were plated on fibronectin and the dynamics of paxillin were analysed using time-lapse TIRF microscopy. Inserts show enlarged FAs. Scale bar, 20 μm. (**E**) Quantification of the FA assembly and disassembly rate constants in MDA-MB-231 cells that express talin2 shRNAs or empty pLKO.1 vector. Quantifications are expressed as mean ± S.E.M. of 60 FAs from 12 cells.

**Figure 2:**
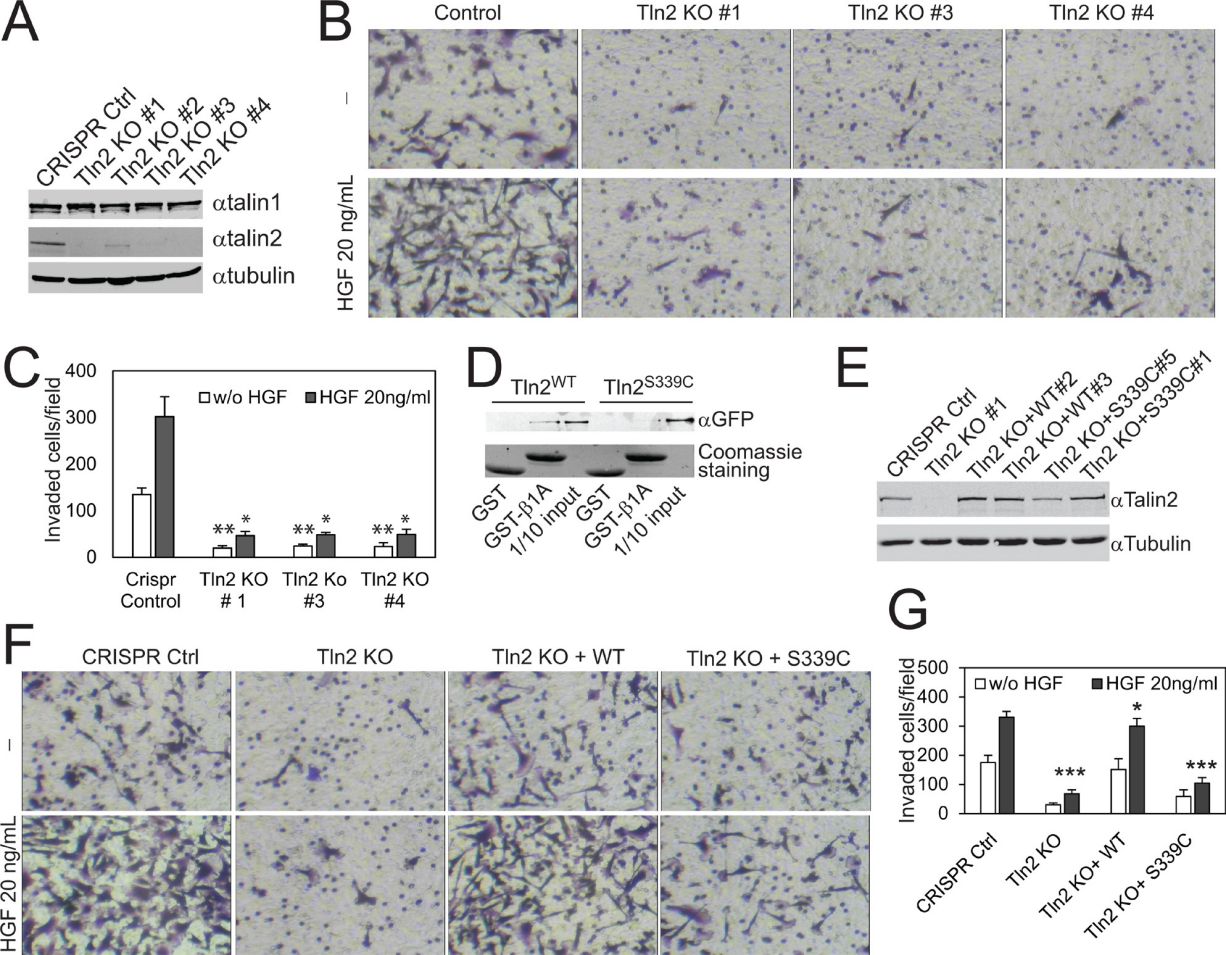
Strong binding of talin2 to integrins is required for the invasion of MDA-MB-231 cells (**A**) Endogenous talin1 and talin2 in CRISPR vector-transfected and talin2-KO MDA-MB-231 cells. (**B**) Ablation of talin2 inhibited the invasion of MDA-MB-231 cells. HGF (20 ng/ml) was added to lower chambers where indicated. (**C**) Quantification of Experiment “B”. Data are presented as mean ± SEM from three independent experiments. *t*-test, ^*^P < 0.05, ^**^P < 0.01, compared to CRISPR control. (**D**) Binding of full-length EGFP-talin2^WT^ or –talin2^S339C^ to β1A-integrin tails measured by GST pull-down assays. The EGFP fusion proteins of talin mutants were transiently expressed in CHO-K1 cells. (**E**) Stable expression of EGFP-talin2^WT^ and -talin2^S339C^ in talin2-null MDA-MB-231 cells using CRISPR. (**F**) Talin2-null MDA-MB-231 cells that express EGFP-talin2^WT^ or –talin2^S339C^ were examined for their Matrigel invasive capacities, using CRISPR vector-infected cells and talin2-null cells as controls. (**G**) Quantification of Experiment “E”. Data are presented as mean ± SEM from three independent experiments. *t*-test, ^*^P < 0.05 and ^***^P < 0.001, compared to CRISPR control.

To examine the role of talin2 in cell migration, endogenous talin2 in MDA-MB-231 cells were depleted by expressing talin2 shRNA (Figure 1A). Cell migration was determined by time-lapse cell migration assays, as described previously [27]. Depletion of talin2 significantly suppressed the migration of MDA-MB-231 cells (Figure 1B), by inhibiting directionality and velocity (Figure 1C). Depletion of talin1 also inhibited cell migration. However, talin1 affected velocity more than directionality, whereas talin2 functioned oppositely (Data not shown). To further examine the biological function of talin2, we used CRISPR/Cas9 to knockout talin2 from MDA-MB-231 cells. Ablation of talin2 was achieved by infecting the cells with lentiviruses that express Cas9 and talin2 gRNAs. Talin2 was completely ablated in clones #1, #3 and #4, while was partially in clone #2 (Figure 2A). Thus, we chose clones #1, #3, and #4 for further experiments. We examined the role of talin2 in cell migration using transwell migration assays. Ablation of talin2 significantly inhibited cell migration in the absence of a growth factor chemoattractant, whereas had no effect on EGF-induced cell migration (20 ng/ml EGF in lower chambers; Supplementary Figure 1). These results suggest that talin2 has distinct roles in cell migration under different conditions.

Because FA dynamics is crucial for cell migration, we examined the role of talin2 in FA dynamics. MDA-MB-231 cells that stably express DsRed-paxillin were infected with lentiviruses that express talin2 shRNA. FA assembly and disassembly were determined as we described previously [13, 27]. As shown in Figure 1D and 1E, depletion of talin2 significantly inhibited both FA assembly/disassembly rates in MDA-MB-231 cells, suggesting that talin2 regulates cell migration by modulating FA dynamics.

To ascertain the role of talin2 in breast cancer cell invasion, the invasion of talin2 knockout (KO) cells was measured by examining the functional capacities of the cells penetrating through transwell filters coated with 0.35 mg/ml Matrigel, using cells infected with empty LentiCrispr vector as a control. Ablation of endogenous talin2 significantly inhibited the basal (without growth factors) as well as HGF-stimulated invasion of MDA-MB-231 cells (Figure 2B, 2C). Depletion of talin2 by using shRNAs also inhibited the invasion of MDA-MB-468 and MDA-MB-435S cells (Supplementary Figure 2).

Previously, we demonstrated that substitution of S339 with Cys caused significant reduction in the binding of the talin2 head domain to β-integrin tails [25]. We examined whether the mutation also inhibits the binding of the full-length talin2 to β-integrins. As shown in Figure 2D, substitution of S339 with Cys also resulted in significant reduction in the binding of the full-length talin2 to β1A-integrin tail.

To determine the essential role of the talin2-β-integrin interaction in cell invasion, EGFP-talin2^WT^ and -talin2^S339C^ were re-expressed in talin2-null MDA-MB-231 cells, respectively (Figure 2E). The invasive capacities of these cells toward Matrigel were tested, using talin2-null cells and CRISPR vector cells as controls. Expression of EGFP-talin2^WT^ in talin2-null cells significantly rescued the basal and HGF-stimulated cell invasion (Figure 2F, 2G). Expression of EGFP-talin2^S339C^ had no effect on basal invasion, but partially rescued HGF-stimulated invasion (*P* < 0.05 KO vs S339C), suggesting that a strong binding of talin2 to integrins is required for cell invasion.

**Figure 3:**
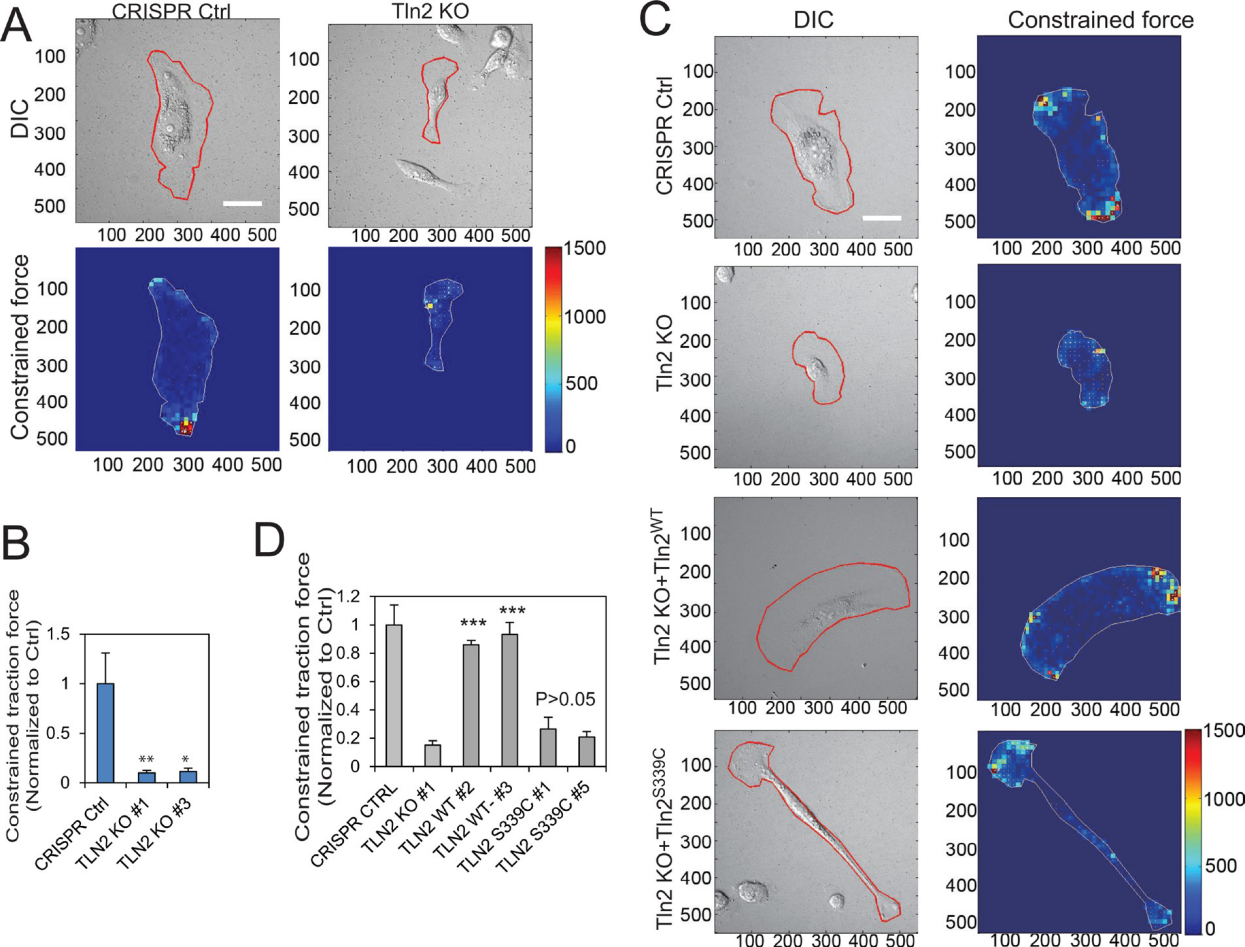
Strong binding of talin2 to β-integrin tails is required for traction force generation (**A**) Effects of talin2 KO on traction force generation in MDA-MB-231 cells. Scale bar, 30 μm. (**B**) Quantitative constrained traction force in talin2-ablated MDA-MB-231 cells, using cells carrying CRISPR vector as a control. Data are presented as mean ± SEM of at least 20 cells from each group. *t*-test, ^*^P < 0.05 and ^**^P < 0.01, compared to CRISPR control. (**C**) Re-expression of talin2^WT^ in talin2-null MDA-MB-231 cells restored their traction force generation, but that of talin2^S339C^ did not. Talin2-null MDA-MB-231 cells that express EGFP-talin2^WT^ or –talin2^S339C^ were cultured on polyacrylamide gel containing Red FluoSpheres for determining traction force, using CRISPR vector-infected cells and talin2-null cells as controls. Scale bar, 30 μm. (**D**) Quantitative constrained traction force. Data are presented as mean ± SEM of at least 40 cells from each group. *t*-test, ^***^P < 0.001, compared to talin2 KO cells.

Because traction force plays an important role in cell migration and invasion [23, 25, 28], we set out to examine the role of talin2 in traction force generation. To this end, talin2-null MDA-MB-231 cells were plated on the fibronectin-conjugated polyacrylamide gels containing Red Fluospheres (Life Technologies), using cells carrying empty CRISPR vector as a control. Traction force was measured by a Nikon A1 confocal microscope equipped with a CO_2_ incubator system, and was analyzed using the method of Butler et al. Ablation of talin2 almost abolished the traction force generation in MDA-MB-231 cells (Figure 3A, 3B). To know whether a strong talin2-β-integrin interaction is required for traction force generation, talin2-null MDA-MB-231 cells that express EGFP-talin2^WT^ or –talin2^S339C^ were plated on the gels containing Red FluoSpheres, and traction force was measured using talin2-null cells and CRISPR vector cells as controls. Expression of EGFP-talin2WT in talin2-null MDA-MB-231 cells restored more than 85% of traction force, whereas that of EGFP-talin2^S339C^, which has reduced affinity to β-integrins, had little effect (Figure 3C, 3D). This result suggests that a strong binding of talin2 to β-integrins is required for traction force generation.

**Figure 4:**
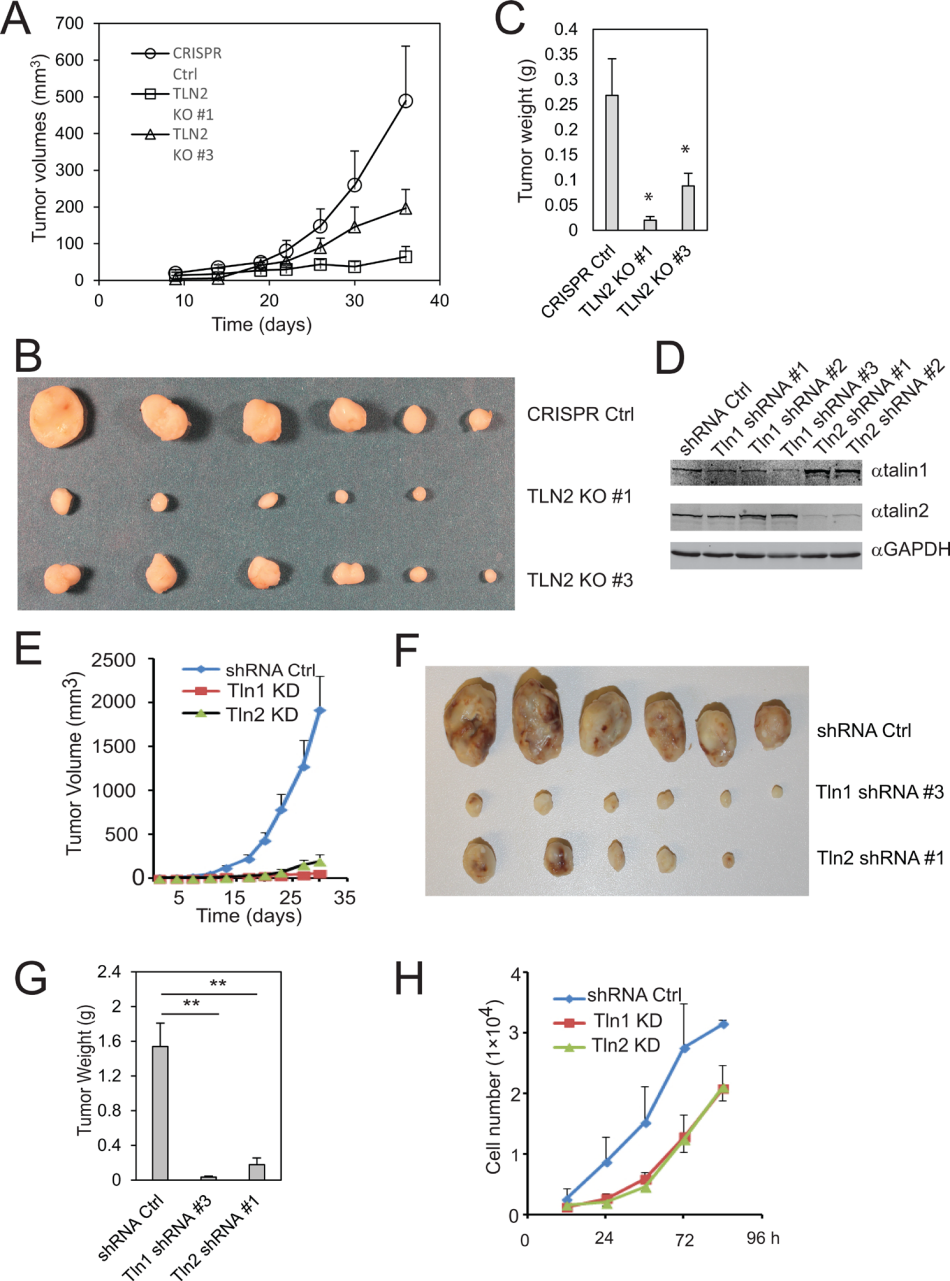
Talin2 is indispensable for tumor growth in a xenograft model of human breast cancer (**A**–**C**), Talin2 KO suppressed breast cancer tumorigenesis. Talin2 KO MDA-MB-231 cells (1 × 10^6^) were injected subcutaneously to female NCR-NU mice (five to six weeks old), using CRISPR vector cells as a control. Tumor size was measured when indicated with calipers, and tumor volumes were calculated and plotted (A). After 36 days, mice were euthanized. Tumors were removed, fixed and photographed (B). Tumor weights were measured with a balance with 0.001 g readability (C). (**D**) MDA-MB-231 cells were infected with lentiviruses that express talin1 or talin2 shRNAs, using empty pLKO.1 vector as a control. (**E**–**G**) Both talin1 and talin2 KD inhibited tumor growth. Talin1 or talin2 KD MDA-MB-231 cells (1 × 10^6^) were injected to female NCR-NU mice (five to six weeks old), using pLKO.1 vector cells as a control. Tumor volumes were calculated and plotted (E). After 30 days, mice were euthanized. Tumors were removed, fixed and photographed (F). Tumor weights were measured (G). (**H**) Growth curves of talin1 and talin2 KD cells, compared to pLKO.1 vector cells.

To study the role of talin2 in tumorigenesis, talin2-null MDA-MB-231 cells were injected into female NCRNU mice (Taconic) by subcutaneous injection, using cells that were infected with empty LentiCrispr vector as a control. Tumor volumes were measured at 3–7 day intervals. As shown in Figure 4A, tumor volumes from mice injected with talin2-null cells were significantly smaller than those from mice injected with the cells transfected with empty vector. At 36 days after injection, mice were euthanized, and tumors were removed, photographed and weighted. Talin2 KO significantly inhibited tumor growth (Figure 4B, 4C). The role of talin2 in tumorigenesis was further demonstrated by using talin2 KD cells. To compare the roles of talin1 and talin2 in tumorigenesis, talin1 and talin2 were depleted with talin1 and talin2 shRNAs, respectively (Figure 4D). MDA-MB-231 cells that express talin2 shRNAs were injected into female NCRNU mice by subcutaneous injection, using cells expressing talin1 shRNA and cells transfected with empty pLKO.1 vector as controls. Tumor volumes were measured at 3 day intervals. As shown in Figure 4E, tumor volumes from mice injected with the cells expressing talin2 shRNA were significantly smaller than those injected with the cells transfected with empty vector, and were similar to those injected with the cells expressing talin1 shRNA. At 30 days after injection, mice were euthanized, and tumors were removed, photographed and weighted. Either talin1 or talin2 knockdown (KD) significantly inhibited tumor growth (Figure 4F, 4G). Depletion of talin1 or talin2 also suppressed cell proliferation *in vitro* (Figure 4H). These data suggest that, like talin1, talin2 is also indispensable for breast cancer cell tumorigenesis.

**Figure 5:**
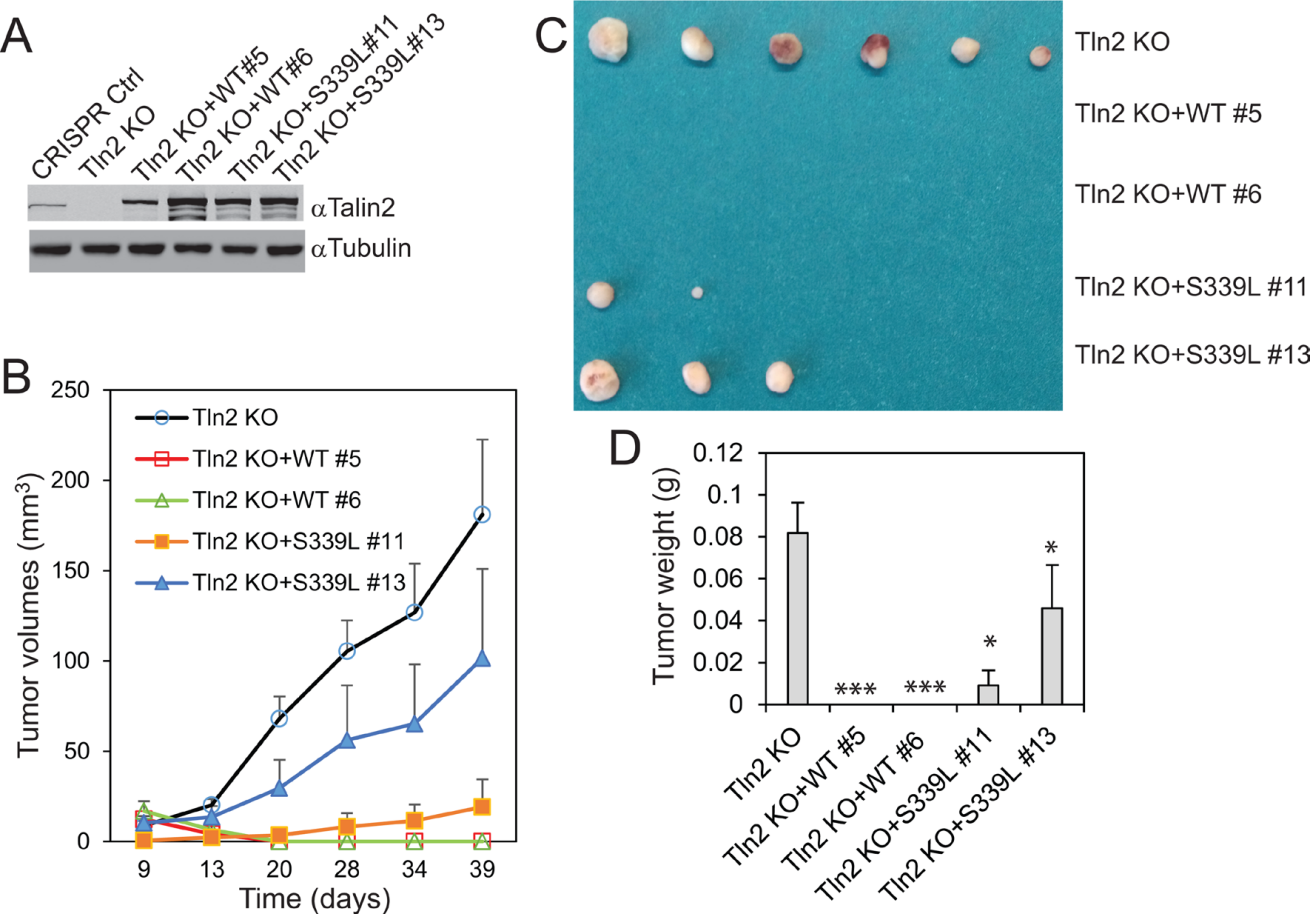
Re-expression of talin2 in talin2-null MDA-MB-231 cells abolished their tumorigenesis (**A**) Stable expression of EGFP-talin2^WT^ and -talin2^S339L^ in talin2-null MDA-MB-231 cells using CRISPR. B-D, Talin2-KO MDA-MB-231 cells that re-express EGFP-talin2^WT^ or -talin2^S339L^ were injected subcutaneously to female NCR-NU mice, using talin2-KO cells as a control. Tumor size was measured when indicated, and tumor volumes were calculated and plotted (**B**). After 39 days, mice were euthanized. Tumors were removed, and photographed (**C**). Tumor weights were measured (**D**).

To determine whether a strong talin2-β-integrin interaction is essential for tumorigenesis, talin2-null MDA-MB-231 cells that re-express EGFP-talin2^WT^ or –talin2^S339L^ were injected into female NCRNU mice (Taconic) by subcutaneous injection, using talin2 null cells as a control. Surprisingly, re-expression of talin2^WT^ in talin2-null cells completely abolished tumor growth, whereas re-expression of talin2^S339L^, an integrin-binding deficient mutant, partially inhibited tumor growth (Figure 5B–5D). This inhibition is probably caused by the high levels of re-expressed talin2^WT^, which are three fold higher than the endogenous talin2 (Figure 5A). These results suggest that exogenous expression of high levels of talin2 can inhibit tumorigenesis more efficiently than talin2 KD (or KO).

**Figure 6:**
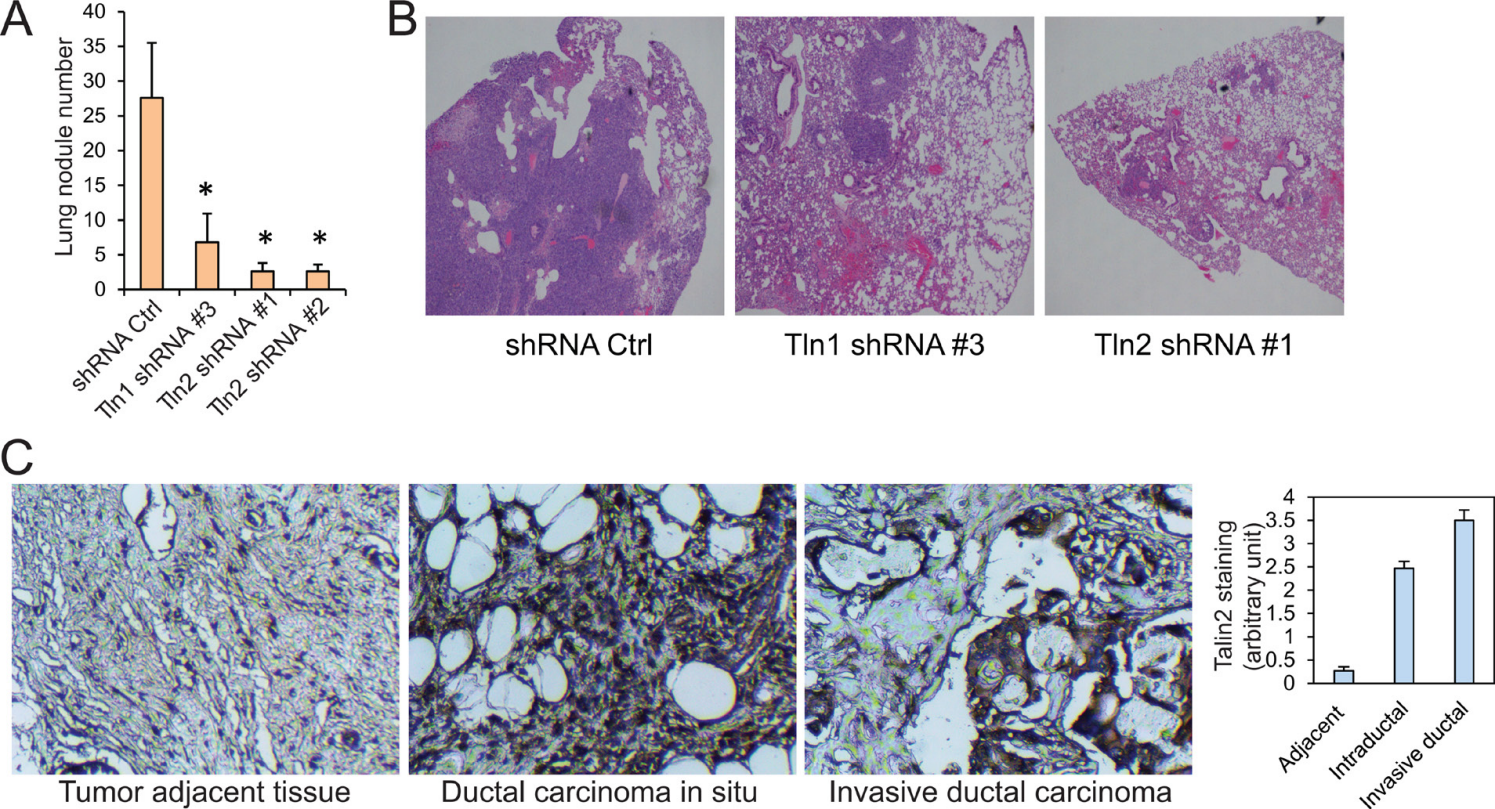
The roles of talin1 and talin2 in the metastasis of MDA-MB-231 cells (**A**) MDA-MB-231 cells that express shRNA control, talin1 shRNA or talin2 shRNA were injected into the tail veins of ICR-SCID mice. After six weeks, lungs were excised, and the numbers of tumor nodules on the lung surface were examined under a dissection microscope and plotted. ^*^P < 0.05. (**B**) Hematoxylin and eosin staining (40×) of paraffin-embedded sections of lung specimens from nude mice implanted with MDA-MB-231 cells that express shRNA control, talin1 shRNA or talin2 shRNA. (**C**) Talin2 staining of human ductal carcinoma *in situ* and invasive ductal carcinoma tissues as well as tumor adjacent tissue.

To know the role of talin2 in breast cancer cell metastasis, MDA-MB-231 cells that express talin2 shRNA or a control shRNA were injected into the tail vein of female NCRNU mice (Taconic), comparing to cells expressing talin1 shRNA. After six weeks, mice were euthanized and tumor nodules on the surface of the lungs were examined microscopically. The lungs from the mice injected with the cells expressing talin1 or talin2 shRNAs had significantly less tumor nodules than those injected with the cells infected with the control shRNA (Figure 6A). Also, hematoxylin and eosin staining showed that the lung sections from the mice injected with cells expressing the control shRNA demonstrated numerous tumor nodules, whereas those from mice injected with cells expressing talin1 or talin2 shRNAs had fewer or no tumor nodules (Figure 6B). These results indicate that both talin1 and talin2 are indispensable for the metastasis of MDA-MB-231 cells.

To examine the possible association of talin2 with breast cancer progression, human breast cancer tissue array slides, including 17 cases of ductal carcinoma *in situ* (DCIS), 32 cases of invasive ductal carcinoma (IDC) and 30 cases of tumor adjacent tissues, were stained for talin2. Immunohistochemical staining was performed as we described previously [29]. Talin2 staining was significantly higher in DCIS and IDC tissues than the staining in the adjacent tissues (*P* < 0.001), and talin2 staining was also higher in IDC tissues than DCIS tissues (*P* < 0.001; Figure 6C). These data suggest that talin2 expression positively correlates with human breast cancer progression.

## DISCUSSION

In breast cancer cells, talin2 mediates traction force generation through its high affinity to β-integrins, thus regulating cell migration, invasion, tumor growth and metastasis. Talin2 is indispensable for tumor growth, although exogenous expression of excessive talin2 can inhibit tumor growth.

We found that depletion of talin2 inhibited the directionality of cell migration in time-lapse migration assays, and that ablation of talin2 suppressed cell migration in transwell migration assays in the absence of a growth factor as a chemoattractant (Figure 1, Supplementary Figure 1). Although the time-lapse migration assays were performed in the presence of EGF, there was no EGF gradient in the assays. Thus, the common factor in these two different assays is lack of EGF gradient, suggesting that talin2 is indispensable for cell migration when there is no signal cue for the directionality. In contrast, talin2 KO had no significant effect on cell migration in the presence of EGF in transwell assays, where the EGF gradient determined the directionality of migration. This result is consistent with previous findings that talin2 deleted mouse embryo fibroblasts showed no obvious defects in the velocity of migration [24]. These results suggest that talin2 regulates cell migration by governing the directionality.

The role of talin2 in determining the directionality of cell migration could be attributed to its effect in traction force (Figure 3), which can regulate the directionality of cell migration [30]. Traction force also regulates focal adhesion dynamics [31–34], a key step in cell migration [20]. This is supported by our findings that talin2 is required for focal adhesion dynamics (Figure 1D, 1E). Moreover, focal adhesions are implicated in determining the directionality of cell migration [35, 36]. Thus, talin2 regulates the directionality of migration by modulating traction force and focal adhesion generation.

We recently reported that talin2 is required for EGF-stimulated invasion of U2 OS human osteosarcoma cells [25]. We show here that ablation of talin2 also inhibited the invasion of MDA-MB-231 cells and traction force generation (Figures 2, 3). Furthermore, re-expression of talin2^WT^ in talin2-null MDA-MB-231 cells rescued cell invasion and traction force generation, whereas re-expression of talin2^S339C^, a β-integrin-binding deficient mutant, did not. These results indicate that a strong interaction between talin2 and β-integrins is essential for traction force generation and cell invasion. Because traction force plays an important role in invadopodia [25], a key cellular structure for invasion, we conclude that talin2-mediated traction force regulates breast cancer cell invasion.

Talin1 knockdown in MDA-MB-231 cells inhibited their tumorigenesis (Figure 4). This is consistent with a previous report in human hepatocellular carcinoma cells [16]. Similar to talin1, talin2 is also indispensable for tumorigenesis in MDA-MB-231 cells. Ablation of talin2 in MDA-MB-231 cells by CRISPR inhibited their tumorigenesis (Figure 4). Similar result was observed in mice injected with talin2 knockdown MDA-MB-231 cells. These results suggest that talin2 is essential for tumor growth.

Surprisingly, re-expression of talin2^WT^ in talin2-null MDA-MB-231 cells did not rescue their tumorigenesis; instead, it completely abolished tumor growth (Figure 5). The inhibition of talin2 is probably associated with the abnormal expression levels of the exogenous talin2, which is much higher than the endogenous. The inhibition is dependent of the interaction of talin2 with β-integrins, because re-expression of talin2^S339L^, a β–integrin-binding deficient mutant, only partially inhibited tumor growth. Thus, raising talin2 expression could be a better strategy to inhibit breast cancer cell tumorigenesis than depleting it, and it is likely that an activator (but not an inhibitor) of the talin2-β-integrin interaction should be developed for cancer treatment.

Nevertheless, the inhibition of talin2 on tumorigenesis seems to be inconsistent with our findings that talin2 staining in DCIS and IDC tissues was significantly higher than the staining in the adjacent tissues (Figure 6). The reason could be that the exogenously expressed talin2 is much higher than the talin2 expression in DCIS and IDC. Because a gene has to collaborate with many other genes in the gene regulatory network to regulate breast cancer growth [37, 38], it is likely that exogenous over-expression of talin2 alone will disrupt the gene regulatory network. Thus, to exogenously express talin2 or its mutants in breast cancer cells for studying tumor growth, a weaker promoter (such as EF-1α) should be selected.

## MATERIALS AND METHODS

### Reagents

Anti-talin1 (clone 97H6) and anti-talin2 (clone 53.8) antibodies were from AbD Serotec. Anti-talin2 rabbit polyclonal antibody (PB9961) was from Boster (Pleasanton, CA). Anti-tubulin (C-terminus) mouse monoclonal antibody (TP1691) was from ECM Biosciences (Versailles, KY). Anti-GAPDH goat polyclonal antibody (A00191) was from Genescript (Piscataway, NJ). pLKO1 lentivirus shRNAs that respectively target talin1 and talin2 were from Sigma. Talin1 shRNA clones are TRCN0000123105 (#1), TRCN0000299020 (#2) and TRCN0000299022 (#3). Talin2 shRNA clones are TRCN0000122990 (#1) and TRCN0000122992 (#2). LentiCRISPRv2 and pSpCas9(BB)-2A-Puro V2.0, which were generated by Dr. Feng Zhang’s Laboratory [39], were from Addgene. Alexa488-labeled gelatin and Red FluoSpheres were from Life Technologies. Dylight 680 labeled goat anti-rabbit IgG (H+L) and Dylight 800-labeled goat anti-mouse IgG (H+L) were from Thermo Scientific. Fibronectin were from Akron Biotech. Growth factor- reduced Matrigel was from BD Bioscience. Pfu Ultra was from Agilent Technologies. Cold Fusion Cloning Kit was from System Biosciences (Palo Alto, CA). Anti-GFP monoclonal antibody and Safectine RU50 transfection kit were purchased from Syd Labs (Malden, MA). Standard VECTASTAIN ABC kit and ImmPACT DAB substrate kit were from Vector Laboratories, Inc. DNA primers were synthesized by Sigma-Aldrich.

### Plasmid construction

The full-length pEGFP-talin2^WT^, pEGFP-talin2^S339C^, pAAVS1-EGFP-talin2^WT^ and pAAVS1-EGFP-talin2^S339C^ were reported previously [25]. The full-length pEGFP-talin2^S339L^ was created by digesting full-length pEGFP-talin2 with BsrG1/EcoRV and ligating the resulting larger fragment with the smaller fragment from pEGFP-talin2_1-449_^S339L^. The full-length pAAVS1-EGFP-talin2^S339L^ was created by digesting full-length pAAVS1-EGFP-talin2^WT^ with BsrG1/EcoRV and ligating the resulting larger fragment with the smaller fragment from pEGFP-talin2_1-449_^S339L^. All plasmids were sequenced by Eurofins MWG Operon (Huntsville, AL).

### Cell culture and transfection

CHO-K1 Chinese hamster ovary cells, MDA-MB-231 human breast cancer cells, and 293T human embryonic kidney cells were from the American Type Culture Collection and were maintained in DMEM medium (Corning Inc.) containing 10% fetal bovine serum (FBS), penicillin (100 U/ml) and streptomycin (100 µg/ml). CHO-K1 and 293T cells were transfected with Safectine RU50 according to the manufacturer’s protocol.

### shRNA knockdown

shRNA plasmids were co-transfected with packaging plasmids (pMDLg/pRRE, pRSV-Rev, and CMV-VSVG) into 293T cells using Safectine RU50 transfection reagent according to the manufacturer’s protocol. The virus particles were applied to overnight cultures of breast cancer cells for infection. Cells that stably express shRNAs were obtained by selecting the infected cells with 1 μg/ml puromycin for 10 days.

### Talin2 re-expression by CRISPR

Talin2-null MDA-MB-231 cells were reported previously [25]. To re-express of talin2 and talin2^S339L^ in talin2-null MDA-MB-231 cells, AAVS1 gRNA was co-transfected with pAAVS1-EGFP-talin2^WT^ and -EGFP-talin2^S339L^ into talin2 null cells. Transfected cells were selected with neomycin. EGFP-positive clones were isolated. The re-expression of EGFP-talin2^WT^ and -EGFP-talin2^S339L^ was examined by western blot.

### Cell migration assays

Cells were treated with trypsin and resuspended in DMEM medium containing 1% FBS and 10 ng/ml EGF, plated at low densities on glass-bottomed dishes (MatTek) coated with 5 µg/ml fibronectin and cultured for three hours in a CO_2_ incubator. Cell motility was measured with a Nikon Biostation IMQ. Cell migration was tracked for six hours; images were recorded every 10 minutes. The movement of individual cell was analyzed with NIS-Elements AR (Nikon) as described previously [27, 29].

To perform Transwell migration assays, Transwell polycarbonate filters were coated with fibronectin (20 µg/ml) for one hour. Cells were trypsinized and washed 3 times with DMEM containing 1% FBS. The cells were resuspended in DMEM containing 1% FBS at a density of 5 × 10^5^ cells/ml. The cell suspensions (100 µl) were seeded into the upper chambers, and 600 µl of DMEM medium containing 1% FBS, and 10 µg/ml fibronectin (and 20 ng/ml EGF where indicated) were added to the lower chambers. The cells were allowed to migrate for 3.5 hours (or as indicated) in a CO2 incubator, fixed, stained and quantitated as described previously [40].

### FA dynamics assays

MDA-MB-231 cells that stably express DsRed-paxillin [27] were infected with lentiviruses that express shRNA control, talin1 or talin2 shRNA and then selected with puromycin. The cells were trypsinized and plated on MatTek dishes (with a glass coverslip at the bottom) that had been precoated with fibronectin (5 µg/ml). The cells were cultured for three hours and TIRF images were taken using the Nikon Eclipse Ti TIRF microscope equipped with a 60×, 1.45 NA objective, CoolSNAP HQ2 CCD camera (Roper Scientific). The temperature, CO_2_ and humidity were maintained by using INU-TIZ-F1 microscope incubation system (Tokai Hit). Images were recorded at 1-minute intervals for a 60-minute period. FA assembly and disassembly rate constants were analyzed as described previously [13, 27, 40].

### Cell invasion assays

Cell invasion was performed as described previously [29, 40]. Briefly, 100 µl of Matrigel (1:30 dilution in serum-free DMEM medium) was added to each Transwell polycarbonate filter and incubated at 37°C for five hours. Cells were trypsinized, washed (three times), and resuspended in DMEM containing 1% FBS at a density of 5 × 10^5^ cells/ml. The cell suspensions (100 µl) were seeded into the upper chambers, and 600 µl of DMEM medium containing 1% FBS, 20 ng/ml HGF and 10 µg/ml fibronectin were added to the lower chambers. The cells were allowed to invade for 10 hours (or as indicated) in a CO2 incubator, fixed, stained and quantitated as described previously [40].

### Traction force measurement

Traction force was measured as described previously [23, 25]. Briefly, glass-bottom dishes were silanized using silane, and activated using glutaraldehyde. Forty µL of gel containing acrylamide (6%), bis-acrylamide (0.75%), ammonium persulfate, TEMED, and FluoSpheres^®^ carboxylate-modified beads (diameter 0.2 μm, 1:85 dilution by volume) was added to the dishes and covered by a coverslip. The coverslip was removed, and gels were activated with sulfo-SANPAH under UVA exposure and then conjugated with fibronectin (0.1 mg/ml). Cells were plated on the gels and traction force was measured as described previously [41], using an A1 confocal microscope in Lexington VA Medical Center.

### Tumor growth assays

Female NCR-NU mice (Taconic, six–seven weeks old) were maintained and treated under pathogen-free conditions. MDA-MB-231 cells (1 × 10^6^ cells/mouse) were injected subcutaneously to female NCR-NU mice. The injected mice were maintained under standard humane conditions. Tumor volume (V) was measured with calipers periodically and calculated by using formula V = 0.5 × LW2 (L, Length; and W, width). Mice were euthanized after 30–40 days. Tumors were removed, weighed and photographed.

### Tumor metastasis assays

Tumor metastasis was performed as described previously [27]. Briefly, female ICR-SCID mice (Taconic) were maintained and treated under pathogen-free conditions. Talin1 or talin2 KD MDA-MB-231 cells (1 × 10^6^ cells/mouse) were injected into the tail vein of mice (six–seven weeks old), using shRNA vector cells as a control. After six weeks, mice were euthanized and lungs were removed and photographed. Tumor nodules present on the surface of lungs were examined under a dissection microscope or detected in paraffin-embedded sections stained with hematoxylin and eosin.

### Immunohistochemical staining

Breast cancer tissue arrays, with stage, grade and normal breast tissue, were purchased from US BioMax. The slides with tissue arrays were deparaffinized with Xylene, dehydrated through three alcohol changes, and performed antigen retrieval in 0.1 M citrate buffer (pH 6.0). The slides were treated with hydrogen peroxide and then blocked with goat serum. The slides were incubated with an affinity purified anti-talin2 rabbit polyclonal antibody and then VECTASTAIN ABC kit (Vector Laboratories, Inc). The slides were visualized by incubating with a DAB substrate kit from the same company. The correlation between talin2 expression and tumor progression was scored and statistically analyzed.

## SUPPLEMENTARY MATERIALS FIGURES


