# Supplementary material for: The role of talin2 in breast cancer tumorigenesis and metastasis
Source: Oncotarget. 2017 Nov 6;8(63):106876–87. doi: 10.18632/oncotarget.22449 (PMC5739781; doi:10.18632/oncotarget.22449)
Supplement: Supplementary file 1 [file oncotarget-08-106876-s001.pdf]

The role of talin2 in breast cancer tumorigenesis and metastasis

SUPPLEMENTARY MATERIALS

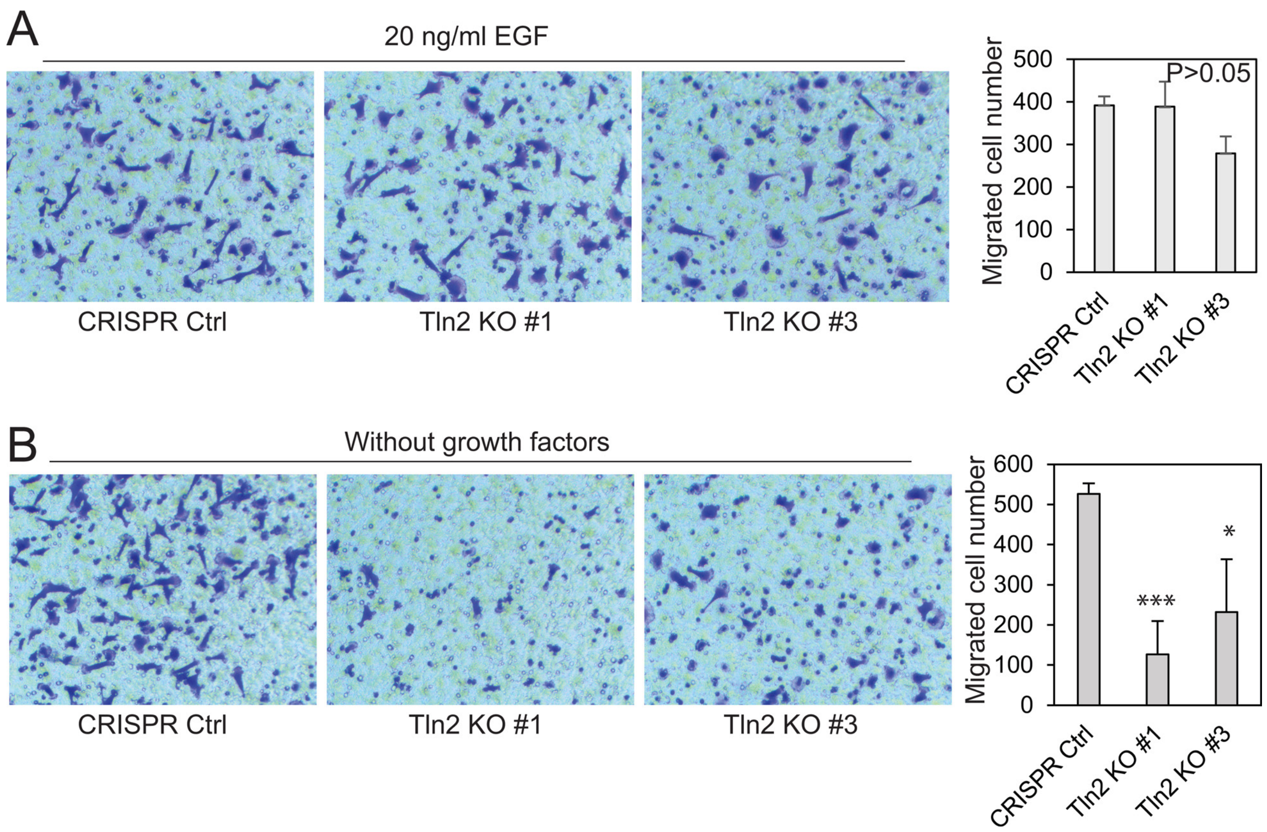

Supplementary Figure 1: The role of talin2 in cell migration, as determined by Transwell migration assays. (**A**) Migration of talin2-ablated MDA-MB-231 cells with 10 µg/ml fibronectin and 20 ng/ml EGF in lower chambers, using the cells infected with empty LentiCrispr vector as a control. Data are presented as mean ± SEM of three independent experiments. (**B**) Migration of talin2-ablated MDA-MB-231 cells with 10 µg/ml fibronectin in lower chambers. Data are presented as mean ± SEM of five independent experiments. $^{*}P < 0.05$, $^{***}P < 0.001$.

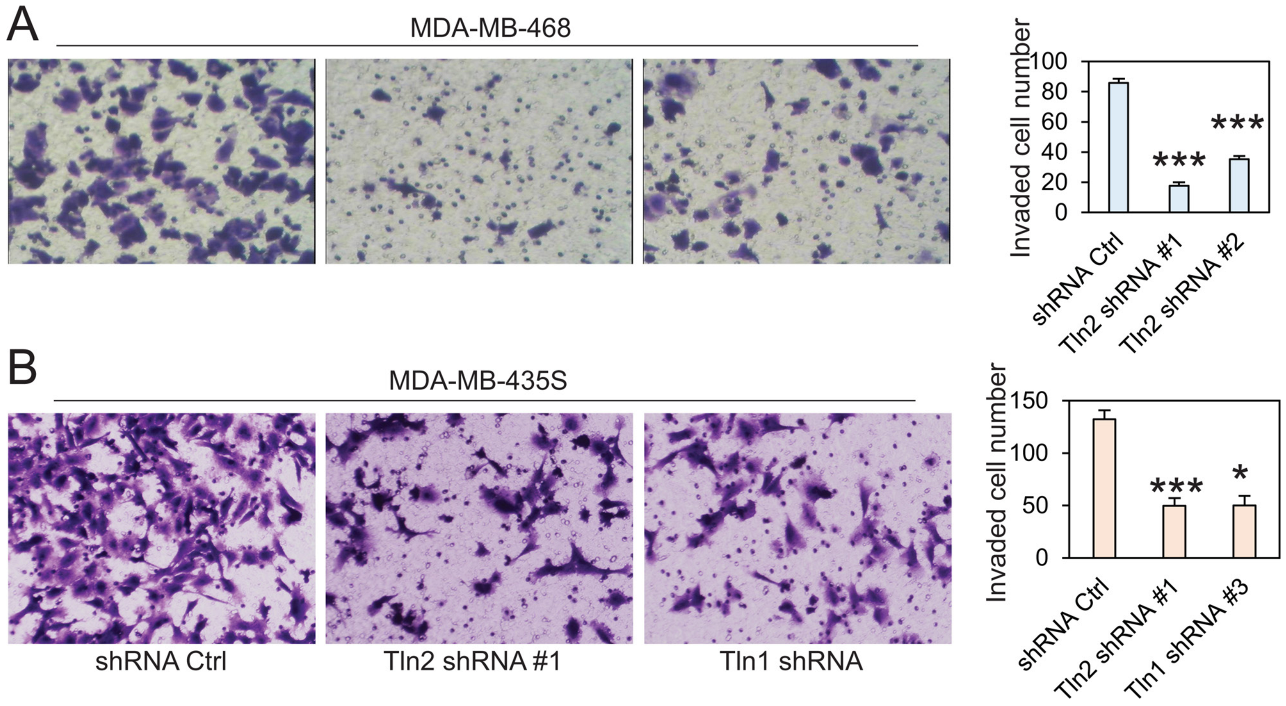

Supplementary Figure 2: The role of talin2 in the invasion of MDA-MB-468 and MDA-MB-435s. (**A**) Depletion of talin2 using shRNAs inhibited the invasion of MDA-MB-468 cells. 10 µg/ml fibronectin and 10% FBS were added to lower chambers, using the cells infected with empty pLKO.1 vector as a control. Data are presented as mean ± SEM of three independent experiments. $^{***}P < 0.001$. (**B**) Depletion of talin1 and talin2 inhibited the invasion of MDA-MB-435s cells. 10 µg/ml collagen and 10% FBS were in lower chambers. Data are presented as mean ± SEM of three independent experiments. $^{*}P < 0.05$, $^{***}P < 0.001$.
